# Robust Minimum Divergence Estimation for the Multinomial Circular Logistic Regression Model

**DOI:** 10.3390/e25101422

**Published:** 2023-10-07

**Authors:** Elena Castilla, Abhik Ghosh

**Affiliations:** 1 Departamento de Matematica Aplicada, Rey Juan Carlos University, Mostoles Campus, 28933 Madrid, Spain; 2 Indian Statistical Institute, Kolkata 700108, India; abhik.ghosh@isical.ac.in

**Keywords:** circular regression, robust estimation, density power divergence

## Abstract

Circular data are extremely important in many different contexts of natural and social science, from forestry to sociology, among many others. Since the usual inference procedures based on the maximum likelihood principle are known to be extremely non-robust in the presence of possible data contamination, in this paper, we develop robust estimators for the general class of multinomial circular logistic regression models involving multiple circular covariates. Particularly, we extend the popular density-power-divergence-based estimation approach for this particular set-up and study the asymptotic properties of the resulting estimators. The robustness of the proposed estimators is illustrated through extensive simulation studies and few important real data examples from forest science and meteorology.

## 1. Introduction

One of the most representative examples in the area of directional statistics are circular data, which are characterized as points in the unit circle. Once the origin and the direction of rotation have been fixed, the observations are measured by their direction represented as an angle θ (from 0 to 2π if measured in radians), a unit vector (cosθ,sinθ), or even a complex number with unit modulus, $e^{i\theta }$. These data represent periodic phenomena, such as directions or measurements over time (time of day, month, lunar cycle...). For a detailed survey of directional statistics we refer to reference [1].

Circular statistics is fast becoming a key instrument in many fields because of its applicability. In biology, for example, it has been applied to the study of avian migration routes [2,3,4] or animal movement [5,6], and to the analysis of mammalian circadian timekeeping [7]. Other examples include leaf inclination angles [8,9]. In atmospheric sciences, circular statistics has been widely applied to the analysis of wind directions [8,9,10,11]. Following this idea, ref. [12] used circular data in the study of wind and solar energy. Other examples include medicine [13] or astrophysics [14]. Circular data are also found in social sciences, such as policy making [15], sociology [16], economics [17] or criminology [9,18].

There are many approaches to model circular data that model the data using a circular distribution. The simplest one is the circular uniform distribution, which assigns equal probabilities to all the points of the circumference. The von Misses (vM) distribution is among the oldest and most used circular distributions. Originally introduced in reference [19] and deeply analyzed in reference [20], the vM distribution has been applied in numerous contexts [15,21,22]. In recent years, some extensions or alternatives to vM distributions have been developed, among which the spherical normal (SN) distribution [23,24,25] has become quite popular.

When we have additional covariates to explain the variations or predict the response data, appropriate regression models involving circular variables have been developed in recent years. The circular–circular regression model relates a circular response variable with a circular explanatory variable [26], while circular–linear regression relates a circular response variable linearly with a vector of given covariates; see, e.g., reference [27] and references therein. On the other hand, the linear–circular regression [28] assumes a linear response variable and circular explanatory variables. The circular logistic regression, introduced by reference [29] and analyzed by references [8,30,31] among others, relates a binary response variable and one circular covariate. Recently, Ref. [9] extended this model to a multinomial response, in the so-called multinomial circular logistic regression (MCLR). The MCLR is an adaptation of the classical multinomial logistic regression (MLR) model, where covariates are not assumed to be circular [32]. Usually the maximum likelihood estimator (MLE) is used to perform inferences for such circular regression models, which is known to be asymptotically most efficient but highly non-robust under possible data contamination [33].

In this paper, we develop robust inference for the general class of MCLR models allowing multiple circular covariates. In particular, a new family of estimators, the minimum density power divergence (DPD) estimators, are defined as a generalization of the classical MLE and its asymptotic properties are studied. An extensive simulation study illustrates the improved robust performance of the proposed method over the existing MLE. The applicability and importance of our proposal is further justified through a few interesting real data examples. Finally, the paper ends with some concluding remarks and future direction for work.

## 2. Model Description

Let us consider that our response variable η has d+1 categories, η∈{1,⋯,d+1}, and that it depends on *k* circular explanatory variables $u={\left(u_{1},\cdots ,u_{k}\right)}^{T}$. If $\pi_{j}\left(\beta \right)$ denotes the probability that η belongs to the *j*-th category, the multinomial circular logistic regression model is given by

(1) $$\begin{array}{cc} \pi_{j}\left(\beta |u\right) & =\frac{\exp\left\{\beta_{j0}+\sum_{l=1}^{k}\left[\beta_{jl}^{\left(1\right)}\cos u_{l}+\beta_{jl}^{\left(2\right)}\sin u_{l}\right]\right\}}{1+\sum_{s=1}^{d}\exp\left\{\beta_{s0}+\sum_{l=1}^{k}\left[\beta_{sl}^{\left(1\right)}\cos u_{l}+\beta_{sl}^{\left(2\right)}\sin u_{l}\right]\right\}}\\ \; & =\frac{\exp\{\beta_{j0}+\beta_{j1}^{\left(1\right)}\cos u_{1}+\beta_{j1}^{\left(2\right)}\sin u_{1}+\cdots +\beta_{jk}^{\left(1\right)}\cos u_{k}+\beta_{jk}^{\left(2\right)}\sin u_{k}\}}{1+\sum_{s=1}^{d}\exp\left\{\beta_{s0}+\beta_{s1}^{\left(1\right)}\cos u_{1}+\beta_{s1}^{\left(2\right)}\sin u_{1}+\cdots +\beta_{sk}^{\left(1\right)}\cos u_{k}+\beta_{sk}^{\left(2\right)}\sin u_{k}\right\}}, \end{array}$$

for j=1,⋯,d, and $\pi_{d+1}\left(\beta |u\right)=1-\sum_{j=1}^{d}\pi_{j}\left(\beta |u\right)$. Here, $\beta_{j}={\left(\beta_{j0},\beta_{j1}^{\left(1\right)},\beta_{j1}^{\left(2\right)},\cdots ,\beta_{jk}^{\left(1\right)},\beta_{jk}^{\left(2\right)}\right)}^{T}$ and $\beta ={\left(\beta_{1}^{T},\cdots ,\beta_{d}^{T}\right)}^{T}\in R^{d(2k+1)}$ is the model parameter vector representing the association between the circular predictors and the multinomial response variable.

Suppose that we observe *n* independent observations divided in *I* covariate patterns, each one with $n_{i}$ observations ($\sum_{i=1}^{I}n_{i}=n$), and an associated vector of explanatory variables $u_{i}={\left(u_{i1},\cdots ,u_{ik}\right)}^{T}$. For the *i*-th covariate pattern, let us consider that the number of response variables that fall in the *j*-th category is denoted by $\nu_{ij}$. For simplicity, we will also denote $\pi_{ij}\left(\beta \right)\equiv \pi_{j}\left(\beta |u_{i}\right)$. Then, the likelihood associated with the model is given by $L\left(\beta \right)\propto \prod_{i=1}^{I}\prod_{j=1}^{d+1}\pi_{ij}{\left(\beta \right)}^{\nu_{ij}},$ and the log-likelihood

(2) $$\begin{array}{c} \ell \left(\beta \right)=\log L\left(\beta \right)\equiv c+\sum_{i=1}^{I}\sum_{j=1}^{d+1}\nu_{ij}\log\pi_{ij}\left(\beta \right), \end{array}$$

for any positive constant *c*; see reference [9] for details.

**Definition 1.**  *Given the multinomial circular logistic regression model (1), the maximum likelihood estimator (MLE) of model parameters **β** is defined as*

$$\begin{array}{c} {\hat{\beta }}_{\mathrm{MLE}}=\arg\max_{\beta \in R^{d(2k+1)}}\ell \left(\beta \right), \end{array}$$

*where ℓ(β) is the log-likelihood based on the observed data as defined in Equation (2).*

Let us introduce the following notation. For the *i*-th covariate pattern, let $\nu_{i}\;=\;{\left(\nu_{i1},\cdots ,\nu_{i(d+1)}\right)}^{T}$ denote the vector of the number of responses within each category, and let $\pi_{i}\left(\beta \right)={\left(\pi_{i1}\left(\beta \right),\cdots ,\pi_{i(d+1)}\left(\beta \right)\right)}^{T}$ denote the vector of model probabilities. With superscript ${}^{*}$ along with a vector (or matrix), we will denote the truncated vector (matrix) obtained by deleting the last value (row) from the initial vector (matrix). Thus, $\nu_{i}^{*}={\left(\nu_{i1},\cdots ,\nu_{id}\right)}^{T}$, $\pi_{i}{\left(\beta \right)}^{*}={\left(\pi_{i1}\left(\beta \right),\cdots ,\pi_{id}\left(\beta \right)\right)}^{T}$ and so on. Let p be a vector and let us denote $\Delta \left(p\right)=diag\left(p\right)-pp^{T}$, where diag(p) denotes the matrix with the entries of p along the diagonal. Finally, let us denote

$$\omega_{i}={\left(1,\cos u_{i1},\sin u_{i1},\cdots ,\cos u_{ik},\sin u_{ik}\right)}^{T},\;i=1,\cdots ,I.$$

Note that, for j=1,⋯,d, we have

(3) $$\begin{array}{c} \frac{\partial \pi_{ij}\left(\beta \right)}{\beta_{j}}=\pi_{ij}\left(\beta \right)\left(1-\pi_{ij}\left(\beta \right)\right)\omega_{i}. \end{array}$$

Computing the derivative of (2) with respect to β and using (3), the estimating equations associated to the MLE are then given, as in the following result. The subsequent result presents the asymptotic distribution of the MLE for the multinomial circular logistic regression model (1); see reference [9] for proofs of both results.

**Proposition 1.**  *Given the multinomial circular logistic regression model (1), the MLE (${\hat{\beta }}_{\mathrm{MLE}}$) of the parameter vector **β** as defined in Definition 1 can be equivalently obtained by solving the following system of estimating equations:*

(4) $$\sum_{i=1}^{I}\left(\nu_{i}^{*}-n_{i}\pi_{i}^{*}\left(\beta \right)\right)⊗\omega_{i}=0_{d(2k+1)},$$

*where* ⊗ *denotes the Kronecker product.*

**Proposition 2.**  *Given the multinomial circular logistic regression model (1), the asymptotic distribution of the MLE of the parameter vector **β**, as defined in Definition 1, is given by*

$$\begin{array}{c} \sqrt{n}\left({\hat{\beta }}_{MLE}-\beta^{0}\right)\overset{L}{\underset{n\to \infty }{⟶}}N\left(0_{d(2k+1)},\Omega^{-1}\left(\beta^{0}\right)\right), \end{array}$$

*where $\beta^{0}$ denotes the true value of **β** and*

$$\Omega \left(\beta \right)=\lim_{n\to \infty }\sum_{i=1}^{I}\frac{n_{i}}{n}\Delta \left(\pi_{i}^{*}\left(\beta \right)\right)⊗\omega_{i}\omega_{i}^{T}.$$

Since the above MLE is known to be highly non-robust (see Section 4 and Section 5 as well for numerical illustrations), we propose a general class of robust minimum density power divergence estimator under the MCLR models in the following section. Note that, in this paper, we refer to robust estimators to those insensitive to outliers. While outliers can provide meaningful information concerning potential model misspecification, these may also stem from measurement or input errors. These failed observations may make a large difference in the results of regression analysis when following the classical maximum likelihood procedures [33], and the development of alternative robust estimators becomes necessary. When outliers come from model misspecification, the problem of outliers may also be solved through an adequate modification on the model describing our data. Other types of robustness, for example to model misspecification, are not discussed here.

## 3. The Minimum DPD Estimators under the MCLR Models

The minimum density power divergence estimator (MDPDE), originally introduced by Basu et al. [34] for independent identically distributed (IID) data, has become extremely popular as a robust generalization of the classical MLE that produces desired robustness under possible data contamination without significant loss in efficiency under clean data. The MDPDE is obtained by minimizing a suitable estimate of a density-based statistical divergence, namely the DPD, between the observed data and the model density. In particular, if we observe IID data $X_{1},\ldots ,X_{n}$ from a population having true (unknown) density *g*, that we model by a family of parametric densities $f_{\theta }$ with $\theta \in \Theta \subseteq R^{p}$, then the MDPDE of the unknown parameter θ is to be obtained by minimizing an estimate of the DPD between *g* and $f_{\theta }$, which is defined as [34]

$$d_{\alpha }\left(g,f_{\theta }\right)=\int f_{\theta }^{1+\alpha }-\left(1+\frac{1}{\alpha }\right)\int f_{\theta }^{\alpha }g+\frac{1}{\alpha }\int g^{1+\alpha },$$

where α>0 is a robustness tuning parameter that controls the trade-off between robustness and efficiency. Since the third term in the form of the DPD does not depend on the parameter of interest (θ), estimating the second term using the empirical distribution function based on the observed data $X_{1},\ldots ,X_{n}$, we get the final MDPDE objective function to be minimized as given by

(5) $$\begin{array}{c} H_{n,\alpha }\left(\theta \right)=\int f_{\theta }^{1+\alpha }-\left(1+\frac{1}{\alpha }\right)\frac{1}{n}\sum_{i=1}^{n}f_{\theta }^{\alpha }\left(X_{i}\right)+\frac{1}{\alpha }. \end{array}$$

As α→0, the DPD measure coincides, in a limiting sense, to the Kullback–Leibler divergence and the associated minimizer (the MDPDE at α=0) is then nothing but the MLE, the most efficient but highly non-robust MLE. This can also be seen by noting that, as α→0, $H_{n,\alpha }\left(\theta \right)$ converges to the negative log-likelihood (ensured by the addition of the constant 1/α). Thus, the MDPDEs provide increased robustness at a cost of slight loss in efficiency as α increases. Please refer to references [34,35] for more details and examples.

Due to various favorable properties of the MDPDE, it has now been extended to several important complex data structures and problem set-ups. In particular, the authors of reference [36] extended the definition and properties of the MDPDE for independent but non-homogeneous set-ups, which is later utilized to study the MDPDEs for many different regression models [36,37,38]. Castilla et al. [39,40] have used the same idea to study the MDPDE for MLR models based on data obtained using simple random sampling and complex survey sampling, respectively. In the present set-up of MCLR models also, the observed responses are independent but each follows a different multinomial distribution depending on the given (or observed) value of the circular predictor variables, and so we will define the MDPDE of the parameters of the MCLR model (1) in the line of references [36,39], which is formally presented in the following definition.

**Definition 2.**  *Given the multinomial circular logistic regression model (1), the MDPDE of model parameters **β** with tuning parameter α>0 is defined as*

$$\begin{array}{c} {\hat{\beta }}_{n,\alpha }=\arg\min_{\beta \in R^{d(2k+1)}}H_{n,\alpha }\left(\beta \right), \end{array}$$

*where we now have*

(6) $$\begin{array}{c} H_{n,\alpha }\left(\beta \right)=\frac{1}{n^{1+\alpha }}\sum_{i=1}^{I}\sum_{j=1}^{d+1}\left[n_{i}^{1+\alpha }\pi_{ij}{\left(\beta \right)}^{1+\alpha }-\left(1+\frac{1}{\alpha }\right)\nu_{ij}n_{i}^{\alpha }\pi_{ij}{\left(\beta \right)}^{\alpha }+\frac{1}{\alpha }\nu_{ij}^{1+\alpha }\right]. \end{array}$$

Note that, as α→0, $H_{n,\alpha }\left(\beta \right)$ converges to the symmetric log-likelihood value defined in (2), plus a constant, so that ${\hat{\beta }}_{n,0}$ is nothing but the MLE defined in Definition 1. The MDPDEs thus again provide a robust generalization of the MLE under the MCLR models with increased robustness under data contamination as α increases with only a little loss in efficiency under pure data; see Section 4 and Section 5 for numerical illustrations justifying this claim. For the computation of the MDPDE under the MCLR models, we can either numerically minimize the objective function given in (6) or, alternatively, solve the associated estimating equations given in the following theorem.

**Theorem 1.**  *Given the multinomial circular logistic regression model (1), the MDPDE ${\hat{\beta }}_{n,\alpha }$ of the parameter vector **β** with tuning parameter α≥0, as defined in Definition 2, can be equivalently obtained by solving the following system of estimating equations:*

(7) $$\sum_{i=1}^{I}n_{i}^{\alpha }\left[\tilde{\Delta }\left(\pi_{i}\left(\beta \right)\right){diag}^{\alpha -1}\left(\pi_{i}\left(\beta \right)\right)\left(\nu_{i}-n_{i}\pi_{i}\left(\beta \right)\right)\right]⊗\omega_{i}=0_{d(2k+1)},$$

*where $\tilde{\Delta }\left(\pi_{i}\left(\beta \right)\right)=\left(I_{d}|0_{d\times 1}\right)\Delta \left(\pi_{i}\left(\beta \right)\right)$.*

**Proof.**  The proof follows by the standard differentiation of the objective function $H_{n,\alpha }$ given in (6) with respect to the parameter vector β and using Equation (3). Details are omitted for brevity.    □

It may be noted that the estimating equations of the MDPDE given in (7) coincide exactly with the estimating equations of the MLE in (4) at α=0. So, the above theorem is a generalization of Proposition 1 covering both the cases of the existing MLE and our newly proposed MDPDEs under the MCLR models.

Next, we derive the asymptotic properties of the proposed MDPDE under the MCLR models using the general results from reference [36]. For this purpose, we will assume that Assumptions (A1)–(A7) of reference [36] hold true for our MCLR models as given in (1), which we will refer to as the Ghosh–Basu Conditions. Under these conditions, it can be seen that the MDPDE is asymptotically consistent as n→∞, having a nice asymptotic normal distribution as formally presented in the following theorem.

**Theorem 2.**  *Suppose that the Ghosh–Basu Conditions hold true for the given multinomial circular logistic regression model (1) with the true value of parameter vector **β** being $\beta^{0}$. Then, the asymptotic distribution of the MDPDE ${\hat{\beta }}_{n,\alpha }$ with tuning parameter α≥0, as defined in Definition 2, is given by*

(8) $$\sqrt{n}\left({\hat{\beta }}_{n,\alpha }-\beta^{0}\right)\overset{L}{\underset{n\to \infty }{⟶}}N\left(0_{d(2k+1)},\Psi_{\alpha }^{-1}\left(\beta^{0}\right)\Omega_{\alpha }\left(\beta^{0}\right)\Psi_{\alpha }^{-1}\left(\beta^{0}\right)\right),$$

*where $\Psi_{\alpha }\left(\beta \right)=\lim_{n\to \infty }\Psi_{n,\alpha }\left(\beta \right)$ and $\Omega_{\alpha }\left(\beta \right)=\lim_{n\to \infty }\Omega_{n,\alpha }\left(\beta \right)$ with*

$$\begin{array}{cc} \Psi_{n,\alpha }\left(\beta \right) & =\sum_{i=1}^{I}{\left(\frac{n_{i}}{n}\right)}^{1+\alpha }\tilde{\Delta }\left(\pi_{i}\left(\beta \right)\right){\mathrm{diag}}^{\alpha -1}\left(\pi_{i}\left(\beta \right)\right){\tilde{\Delta }}^{T}\left(\pi_{i}\left(\beta \right)\right)⊗\omega_{i}\omega_{i}^{T},\\ \Omega_{n,\alpha }\left(\beta \right) & =\sum_{i=1}^{I}{\left(\frac{n_{i}}{n}\right)}^{1+\alpha }\tilde{\Delta }\left(\pi_{i}\left(\beta \right)\right){\mathrm{diag}}^{\alpha -1}\left(\pi_{i}\left(\beta \right)\right)\Delta \left(\pi_{i}\left(\beta \right)\right){\mathrm{diag}}^{\alpha -1}\left(\pi_{i}\left(\beta \right)\right){\tilde{\Delta }}^{T}\left(\pi_{i}\left(\beta \right)\right)⊗\omega_{i}\omega_{i}^{T}. \end{array}$$

**Proof.**  The proof follows from the general result of reference [36] in a manner similar to the proof of Theorem 1 in reference [39]. Details are omitted for brevity.    □

**Remark 1.**  *After some algebraic manipulations detailed in Appendix A, it can be seen that $\Psi_{0}\left(\beta \right)=\Omega_{0}\left(\beta \right)=\Omega \left(\beta \right)$, so that Proposition 2 can be recovered directly from Theorem 2 at α=0.*

Theorem 2 is thus applicable, again, for the whole class of MDPDE, including the MLE at α=0, and can be used to obtain the standard error of these estimates under the MCLR models. For this purpose, the asymptotic variance matrix of ${\hat{\beta }}_{n,\alpha }$ can be directly estimated by $n^{-1}\Psi_{n,\alpha }^{-1}\left({\hat{\beta }}_{n,\alpha }\right)\Omega_{n,\alpha }\left({\hat{\beta }}_{n,\alpha }\right)\Psi_{n,\alpha }^{-1}\left(\beta^{0}\right)$, from which the standard errors of the estimates of each component parameter can be obtained by taking the square root of the corresponding diagonal entries of this estimated (asymptotic) variance matrix. This asymptotic variance estimates of the MDPDEs, along with the estimators themselves, can also be used to develop a robust test of statistical hypothesis under the MCLR models in a routine manner.

## 4. Simulation Studies

We consider a simulation scheme with d+1=3 categories and one circular explanatory variable (k=1). The true value of the parameters is taken to be $\beta ={\left(0,2,2,0.2,2.5,1.5\right)}^{T}$. To generate the data, we consider different numbers of covariate patterns (*I*), different sample sizes ($n_{i}$, i=1,⋯,I), and three different generating distributions: the uniform distribution, the vM distribution with mean $60^{\circ }$ and concentration parameter κ=2, and the SN distribution with mean $60^{\circ }$ and concentration parameter κ=6. These samples are generated with libraries circular and Riemann in R (see Appendix B for more details on the circular distributions used here).

The robustness of the proposed estimators is evaluated by introducing 10% outliers in the data. These are artificially classified in the first category, independent from the value of their explanatory variables.

**Figure 1:**
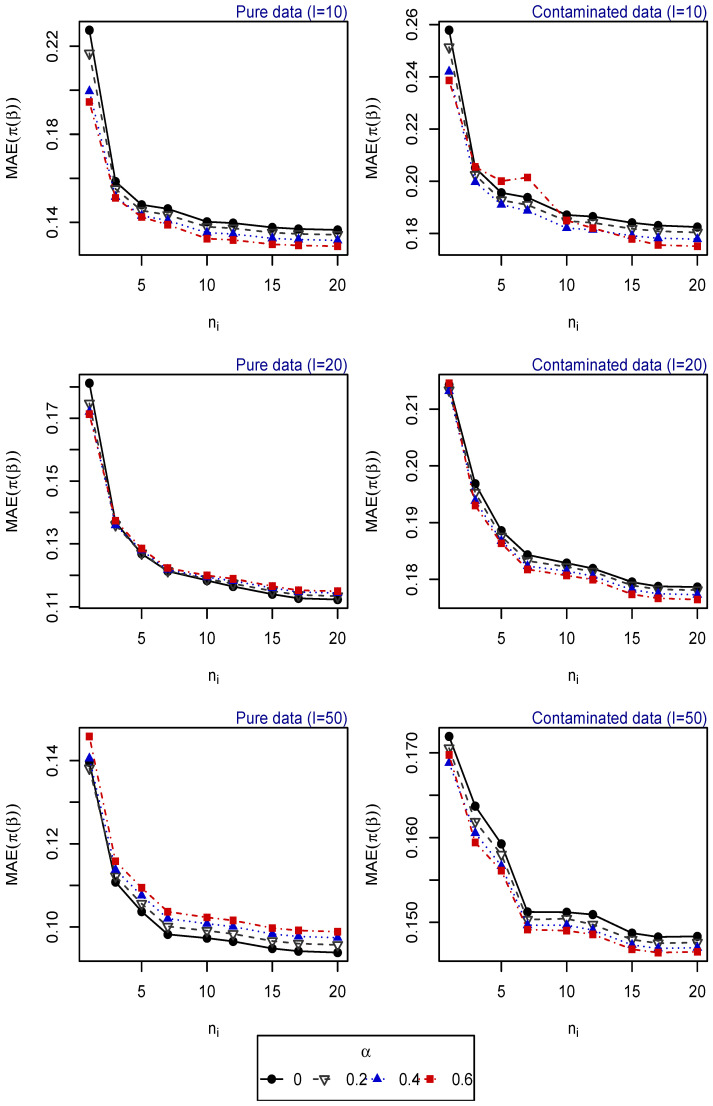
MAE of estimated probabilities when data are generated using uniform distribution for different values of *I* and $n_{i}$, i=1,⋯,I.

**Figure 2:**
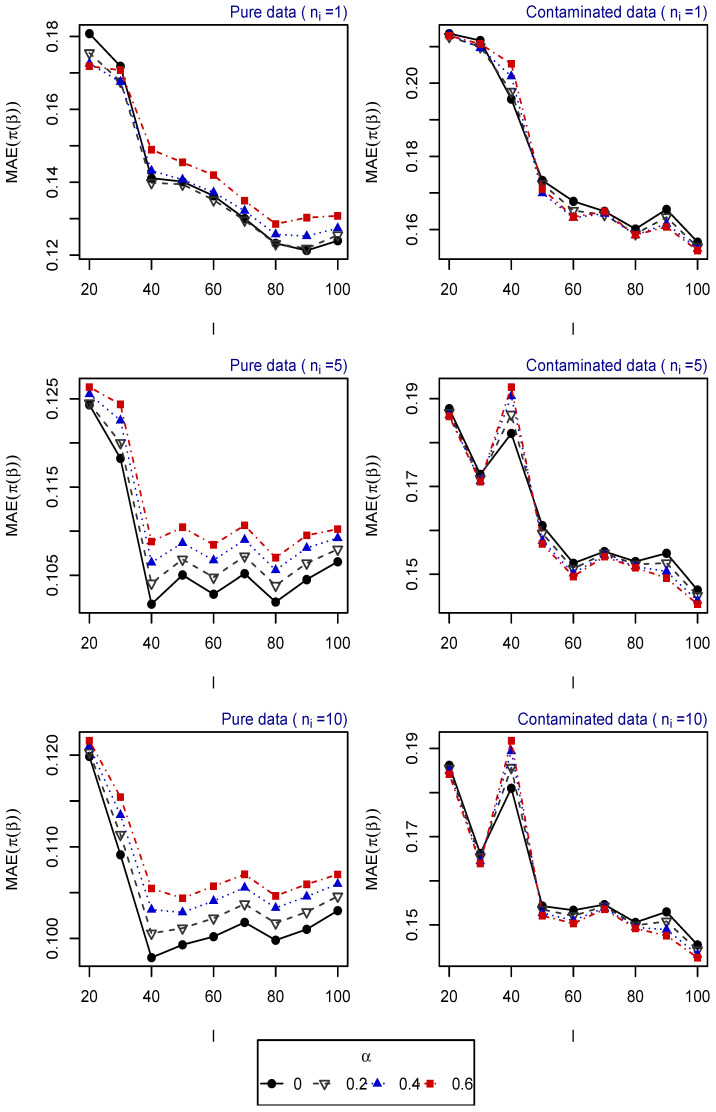
MAE of estimated probabilities when data are generated using uniform distribution for different values of *I* and $n_{i}$, i=1,⋯,I.

**Figure 3:**
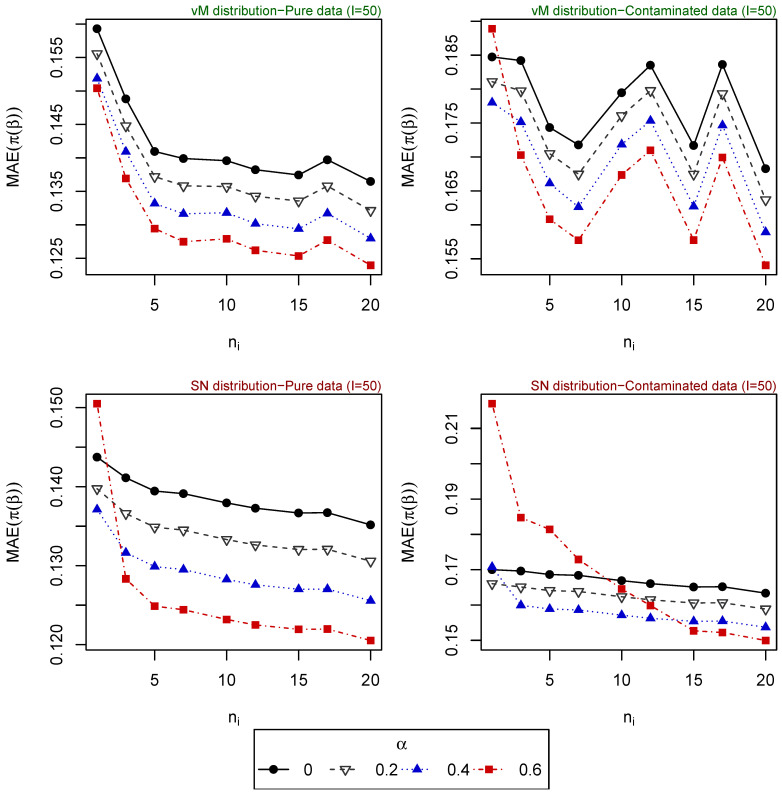
MAE of estimated probabilities when data are generated using vM distribution (above) or SN distribution (below) for I=50 and different values of $n_{i}$, i=1,⋯,I.

**Figure 4:**
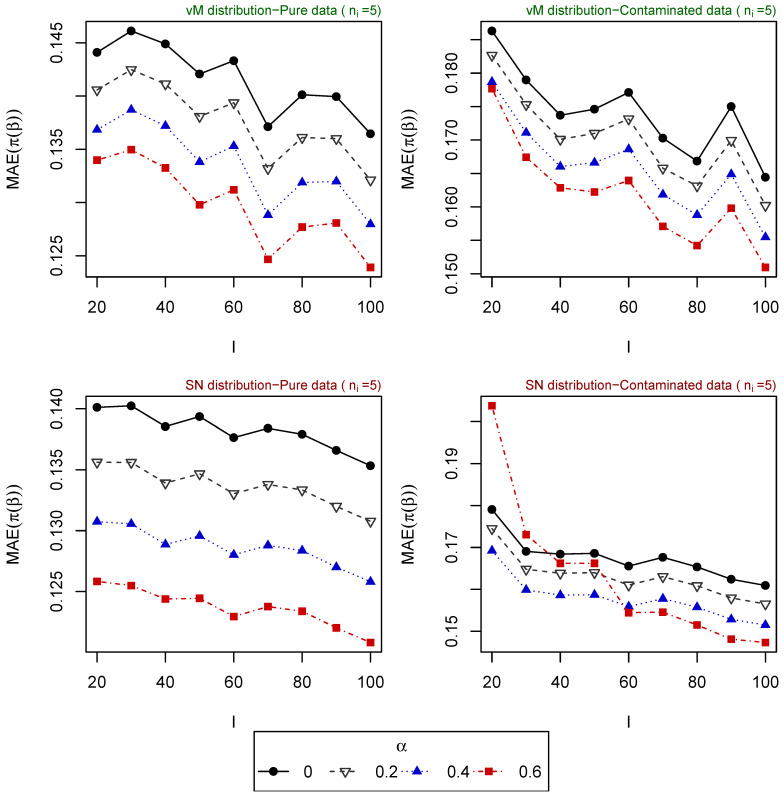
MAE of estimated probabilities when data are generated using vM distribution (above) or SN distribution (below) for $n_{i}=10$, i=5,⋯,I, and different values of *I*.

**Figure 5:**
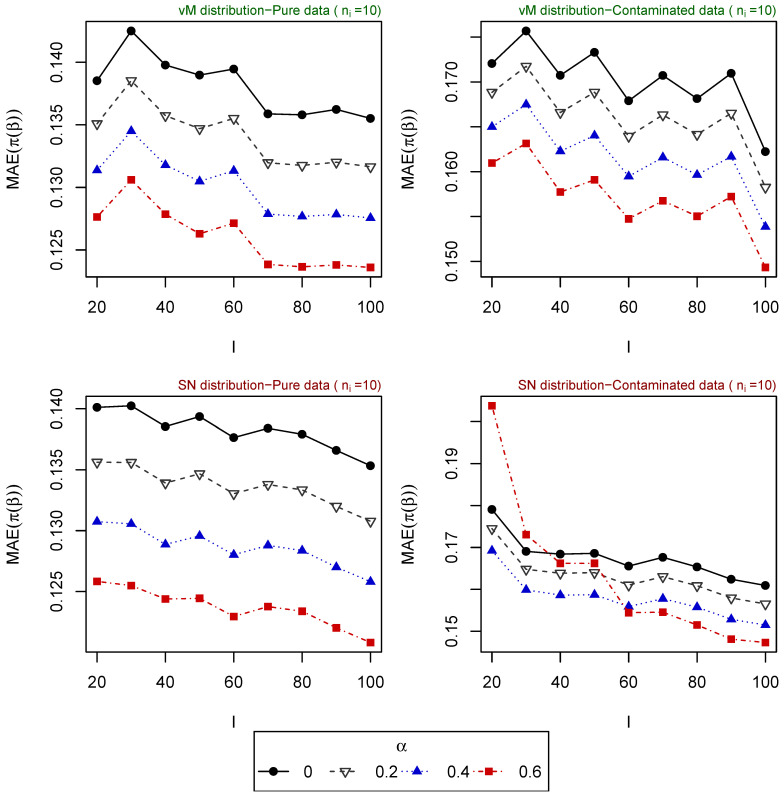
MAE of estimated probabilities when data are generated using vM distribution (above) or SN distribution (below) for $n_{i}=10$, i=1,⋯,I, and different values of *I*.

For 500 replications, the vector of parameters β is estimated for different tuning parameters α, and the mean absolute error (MAE) of the estimated probabilities (obtained based on the estimated vector of parameters) is computed for each one of the following scenarios:

- Scenario 1: I∈{10,20,50}, $n_{i}\in \left\{1,\cdots ,20\right\}$ for i=1,⋯,I. Explanatory variables generated from uniform distribution (Figure 1).
- Scenario 2: I∈{20,⋯,100}, $n_{i}\in \left\{1,5,10\right\}$ for i=1,⋯,I. Explanatory variables generated from uniform distribution (Figure 2).
- Scenario 3: I=50, $n_{i}\in \left\{1,\cdots ,20\right\}$ for i=1,⋯,I. Explanatory variables generated from vM distribution (top of Figure 3).
- Scenario 4: I=50, $n_{i}\in \left\{1,\cdots ,20\right\}$ for i=1,⋯,I. Explanatory variables generated from SN distribution (bottom of Figure 3).
- Scenario 5: I∈{20,⋯,100}, $n_{i}=5$ for i=1,⋯,I. Explanatory variables generated from vM distribution (top of Figure 4).
- Scenario 6: I∈{20,⋯,100}, $n_{i}=5$ for i=1,⋯,I. Explanatory variables generated from SN distribution (bottom of Figure 4).
- Scenario 7: I∈{20,⋯,100}, $n_{i}=10$ for i=1,⋯,I. Explanatory variables generated from vM distribution (top of Figure 5).
- Scenario 8: I∈{20,⋯,100}, $n_{i}=10$ for i=1,⋯,I. Explanatory variables generated from SN distribution (bottom of Figure 5).

The resulting values of the MAEs are plotted in Figure 1, Figure 2, Figure 3, Figure 4 and Figure 5 for each of the above simulation set-ups. An increment on the sample size increases the confidence levels of our estimates, as observed in Figure 1, Figure 2, Figure 3, Figure 4 and Figure 5, where MAE decreases, generally, as *I* or $n_{i}$ increases. It can be seen that MDPDEs generally outperform the MLE when data are contaminated for all the generating distributions and the sample sizes considered. In particular, when vM and SN distributions generate the explanatory variables, the MDPDEs even outperform the MLE when no contamination is introduced in our simulated data.

When data are generated under the uniform distribution, an increment on the tuning parameter α increases the robustness but also decreases the efficiency when pure data are considered. A high value of α may also lead to greater errors when data are generated under vM and SN distributions, both for pure and contaminated data. If we suspect our data are not highly contaminated, a value of α∈(0,0.4] may present a good trade-off between efficiency and robustness. If we detect important outliers in our data, a higher value of α may be preferable. In Section 5, we present some numerical examples which confirm this idea.

## 5. Applications to Real Data

### 5.1. Application to Forest Science

For this application, we use the leaf inclination dataset recorded in reference [41] and analyzed in reference [8,9]. These data contain the leaf inclination angles of 138 plant species. With only this circular variable and applying the circular logistic regression model presented here, we want to classify these species of plants. In particular, we focus on the following examples that have a binary response variable. For each one, we compute the accuracy of the model under different tuning parameters. Contamination in the data is also induced in order to see the effect on our model.

#### 5.1.1. First Example [*Alnus incana* vs. *Alnus glutinosa*]

**Figure 6:**
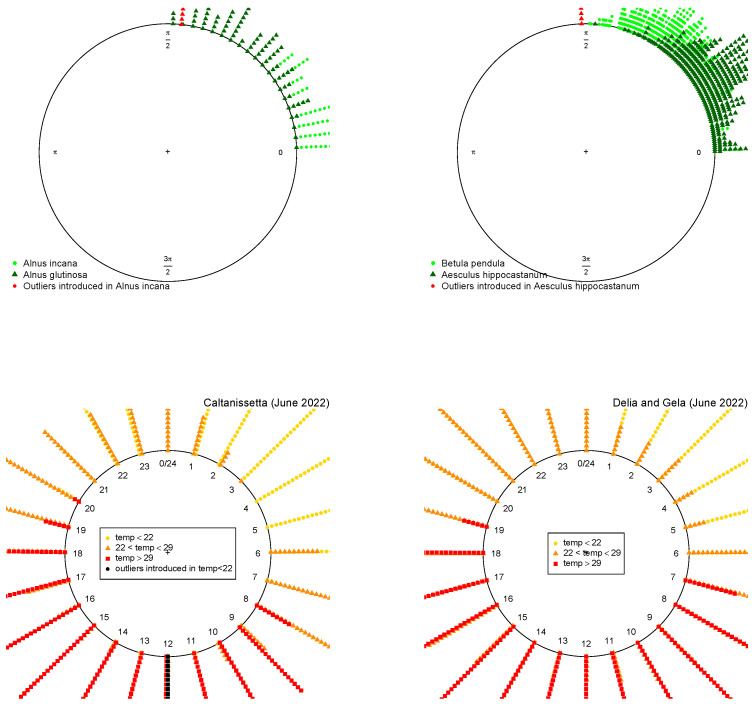
**Above**: Application to forest science (leaf inclinations). **Below**: Application to meteorological science (hours) for original (**left**) and new data (**right**).

These data contain 160 observations of the leaf inclination of *Alnus incana* and *Alnus glutinosa* (80 of each type), which are illustrated in the top left of Figure 6. The circular logistic model with only this covariate has more than 76% of accuracy for the MLE, which is increased by 2% for the proposed MDPDEs (see Table 1). Further, we artificially introduce 16 extreme samples (a 10% of total observations) of *Alnus incana* with null inclination angle, as can be seen in Figure 6; then, the accuracy of all estimations decays but our MDPDEs with α>0 provide significantly higher accuracy than the MLE (Table 1), illustrating their claimed robustness.

#### 5.1.2. Second Example [*Betula pendula* vs. *Aesculus hippocastanum*]

These data, presented in the top right of Figure 6, contain 902 observations of the species *Betula pendula* and *Aesculus hippocastanum*, and were also analyzed in references [8,9]. The circular logistic model with only this covariate has more than 73% of the accuracy with the MLE, which is increased again for the MDPDEs with α>0 (see Table 1). When we manually introduced 45 extreme observations (5% of total observations) of *Aesculus hippocastanum* with null inclination angle, the higher robustness of the proposed MDPDEs are, again, clearly evident from the results presented in Table 1.

### 5.2. Application to Meteorological Science

We now apply the MCLR model to a meteorological dataset, obtained from the “Portale Open Data della Regione Siciliana” (https://dati.regione.sicilia.it/) (accessed on 1 April 2023) containing the temperature of wind at two meters of height in June 2022 in the region of Caltanissetta, a commune capital of the Province of Caltanissetta (Sicilia, Italy), during the whole day with a total of 684 observations. Our response variable is the temperature associated to the hour, divided into three categories: (i) lower than $22^{\circ }$, (ii) between $22^{\circ }$ and $29^{\circ }$ and (iii) greater than $29^{\circ }$. Then, we have balanced data with approximately one third of the observations in each group (See Figure 6). The results of the accuracy of the model are presented in Table 1. As observed, more than 70% of the cases are correctly classified, without a significant difference between estimates. Let us now introduce some outliers to our data. In particular, let us introduce 5% of the low temperatures at 12 noon. The accuracy of MLE and minimum DPD with low values of the tuning parameter decreases significantly, but remains stable for higher values of the tuning parameter.

Finally, we also consider the wind temperatures in the same dates of two other communes in Caltanissetta: Delia and Gela (see Figure 6). If we predict the wind temperature of these 1368 new observations with the model estimated for the first region, we obtain more than 72% accuracy.

## 6. Conclusions

In this paper, we have studied a class of robust minimum divergence estimators, based on the popular density power divergence measure, for the multinational circular logistic regression models, which is extremely important for the circular data frequently occurring in ecological and environmental sciences, among other domains. We have defined the minimum DPD estimators for the multinomial circular logistic regression models as a robust generalization of the classical MLE and derived its asymptotic distribution. The improved performances of the proposed MDPDE under the multinational circular logistic regression models are illustrated numerically with extensive simulation studies and important real-life applications. Our proposed estimators present an increase of up to 6% in accuracy compared to MLE when artificial outliers are introduced in our data sets.

We have also discussed the estimation of the asymptotic variance of the MDPDEs, and hence their standard errors, which can further be used to conduct statistical hypothesis testing robustly under the multinational circular logistic regression models. Along with further study on such hypothesis testing problems, we would also like to extend the MDPDE further for more complex important model set-ups involving circular and general directional data in our future works.

## Figures and Tables

**Table 1:** Accuracy of the multinomial circular regression model for forest and meteorological data.

Dataset→	First Forest Example		Second Forest Example		Meteorological Example		
α↓	**Original**	**Contaminated**	**Original**	**Contaminated**	**Original**	**Contaminated**	**New**
0 (MLE)	0.7625	0.7486	0.7339	0.6705	0.7135	0.6120	0.7208
0.2	0.7812	0.7486	0.7350	0.6727	0.7135	0.6218	0.7208
0.4	0.7812	0.7600	0.7350	0.7223	0.7135	0.6387	0.7208
0.6	0.7812	0.7771	0.7350	0.7307	0.7135	0.6387	0.7208
0.8	0.7812	0.7771	0.7350	0.7381	0.7135	0.6639	0.7208

## Abbreviations

The following abbreviations are used in this manuscript:

DPD	Density Power Divergence
IID	Independent Identically Distributed
MAE	Mean Absolute Error
MCLR	Multinomial Circular Logistic Regression
MDPDE	Minimum Density Power Divergence Estimator
MLE	Maximum Likelihood Estimator
MLR	Multinomial Logistic Regression
vM	Von Mises (distribution)
SN	Spherical Normal (distribution)

## Appendix A. Proof of Remark 1

For each i=1,⋯,I, we have

$$\begin{array}{cc} \; & \tilde{\Delta }\left(\pi_{i}\left(\beta \right)\right){\mathrm{diag}}^{-1}\left(\pi_{i}\left(\beta \right)\right){\tilde{\Delta }}^{T}\left(\pi_{i}\left(\beta \right)\right)\\ \; & =\left(I_{d}|0_{d\times 1}\right)\Delta \left(\pi_{i}\left(\beta \right)\right){\mathrm{diag}}^{-1}\left(\pi_{i}\left(\beta \right)\right)\Delta \left(\pi_{i}\left(\beta \right)\right){\left(I_{d}|0_{d\times 1}\right)}^{T}\\ \; & =\left(I_{d}|0_{d\times 1}\right)\left[\mathrm{diag}\left(\pi_{i}\left(\beta \right)\right)-\pi_{i}\left(\beta \right)\pi_{i}^{T}\left(\beta \right)\right]{\mathrm{diag}}^{-1}\left(\pi_{i}\left(\beta \right)\right)\\ \; & \;\;\times \left[\mathrm{diag}\left(\pi_{i}\left(\beta \right)\right)-\pi_{i}\left(\beta \right)\pi_{i}^{T}\left(\beta \right)\right]{\left(I_{d}|0_{d\times 1}\right)}^{T}\\ \; & =\left(I_{d}|0_{d\times 1}\right)\left[I_{d+1}-\pi_{i}\left(\beta \right)\pi_{i}^{T}\left(\beta \right){\mathrm{diag}}^{-1}\left(\pi_{i}\left(\beta \right)\right)\right]\\ \; & \;\;\times \left[\mathrm{diag}\left(\pi_{i}\left(\beta \right)\right)-\pi_{i}\left(\beta \right)\pi_{i}^{T}\left(\beta \right)\right]{\left(I_{d}|0_{d\times 1}\right)}^{T}\\ \; & =\left(I_{d}|0_{d\times 1}\right)\left[I_{d+1}-2\pi_{i}\left(\beta \right)\pi_{i}^{T}\left(\beta \right)+\pi_{i}\left(\beta \right)\pi_{i}^{T}\left(\beta \right){\mathrm{diag}}^{-1}\left(\pi_{i}\left(\beta \right)\right)\pi_{i}\left(\beta \right)\pi_{i}^{T}\left(\beta \right)\right]{\left(I_{d}|0_{d\times 1}\right)}^{T}\\ \; & =\left(I_{d}|0_{d\times 1}\right)\left[\mathrm{diag}\left(\pi_{i}\left(\beta \right)\right)-\pi_{i}\left(\beta \right)\pi_{i}^{T}\left(\beta \right)\right]{\left(I_{d}|0_{d\times 1}\right)}^{T}=\tilde{\Delta }\left(\pi_{i}\left(\beta \right)\right){\left(I_{d}|0_{d\times 1}\right)}^{T}=\Delta \left(\pi_{i}^{*}\left(\beta \right)\right). \end{array}$$

Therefore,

$$\begin{array}{cc} \Psi_{0}\left(\beta \right) & =\lim_{n\to \infty }\sum_{i=1}^{I}\left(\frac{n_{i}}{n}\right)\tilde{\Delta }\left(\pi_{i}\left(\beta \right)\right){\mathrm{diag}}^{-1}\left(\pi_{i}\left(\beta \right)\right){\tilde{\Delta }}^{T}\left(\pi_{i}\left(\beta \right)\right)⊗\omega_{i}\omega_{i}^{T}\\ \; & =\lim_{n\to \infty }\sum_{i=1}^{I}\left(\frac{n_{i}}{n}\right)\Delta \left(\pi_{i}^{*}\left(\beta \right)\right)⊗\omega_{i}\omega_{i}^{T}=\Omega \left(\beta \right). \end{array}$$

In a similar manner, $\Omega_{0}\left(\beta \right)=\Omega \left(\beta \right)$.

## Appendix B. Details on Circular Distributions

Let us consider data distribution $x\in S^{1}$ (i.e., $x\in R^{2}$ and $x_{2}=1$). The unifrom distribution assigns equal probability to all points of the circle, with probability density function

$$f_{{\mathrm{Uniform}}_{S^{1}}}\left(x\right)=\frac{1}{2\pi }.$$

On the other hand, for the unit circle, the vM distribution with mean $\mu \in S^{1}$ and concentration $\kappa \in R^{+}$ has probability density function

$$f_{\mathrm{vM}}\left(x;\mu ,\kappa \right)=\frac{1}{2\pi I_{0}\left(\kappa \right)}\exp\left(\mu^{T}x\right),$$

where $I_{0}\left(\kappa \right)$ is the modified Bessel function of the first kind of order 0. The vM distribution is a particular case of the vM–Fisher distribution on the *n*-sphere [20].

Finally, the SN distribution [24] with mean $\mu \in S^{1}$ and concentration $\kappa \in R^{+}$ is defined in the unit circle by

$$f_{\mathrm{SN}}\left(x;\mu ,\kappa \right)=\frac{1}{Z(\kappa )}\exp\left(-\frac{\kappa }{2}d^{2}\left(x,\mu \right)\right),$$

where $d^{2}\left(\cdot ,\cdot \right)$ is the geodesic distance and Z(κ) is a normalizing parameter

$$Z\left(\kappa \right)=\int_{S^{1}}\exp\left(-\frac{\kappa }{2}d^{2}\left(x,\mu \right)\right)dx.$$

Note that, for both vM and SN distribution, the mean μ and concentration κ are measures of direction and concentration, respectively. For small κ both distributions are close to the uniform and, in particular,

$${\mathrm{vM}}_{S^{1}}\left(\mu ,\kappa =0\right)={\mathrm{SN}}_{S^{1}}\left(\mu ,\kappa =0\right)={\mathrm{Uniform}}_{S^{1}}.$$

For more details on these distributions, one may refer to reference [25].
